# Thrombodynamics—A new global hemostasis assay for heparin monitoring in patients under the anticoagulant treatment

**DOI:** 10.1371/journal.pone.0199900

**Published:** 2018-06-28

**Authors:** Anna N. Balandina, Ilya I. Serebriyskiy, Alexander V. Poletaev, Dmitry M. Polokhov, Marina A. Gracheva, Ekaterina M. Koltsova, David M. Vardanyan, Irina A. Taranenko, Alexey Yu. Krylov, Evdokiya S. Urnova, Kirill V. Lobastov, Artem V. Chernyakov, Elena M. Shulutko, Andrey P. Momot, Alexander M. Shulutko, Fazoil I. Ataullakhanov

**Affiliations:** 1 Dmitry Rogachev National Research Center of Pediatric Hematology, Oncology and Immunology, Moscow, Russia; 2 Center for Theoretical Problems of Physicochemical Pharmacology, Moscow, Russia; 3 Russian Laboratory Federation, Moscow, Russia; 4 City Clinical Hospital No. 15, Moscow, Russia; 5 Altay Regional Clinical Hospital, Barnaul, Russia; 6 Federal State Autonomous Educational Institution of Higher Education I.M. Sechenov First Moscow State Medical University, Moscow, Russia; 7 National Research Center for Hematology, Moscow, Russia; 8 City Clinical Hospital No. 24, Moscow, Russia; 9 National Research Center for Hematology, Altay Department, Barnaul, Russia; 10 Moscow State University, Moscow, Russia; 11 Moscow Institute of Physics and Technology, Dolgoprudny, Russia; The University of Tokyo, JAPAN

## Abstract

**Background:**

Heparin therapy and prophylaxis may be accompanied by bleeding and thrombotic complications due to individual responses to treatment. Dosage control based on standard laboratory assays poorly reflects the effect of the therapy. The aim of our work was to compare the heparin sensitivity of new thrombodynamics (TD) assay with sensitivity of other standard and global coagulation tests available to date.

**Study population and methods:**

A total of 296 patients with high risk of venous thromboembolism (deep vein thrombosis (DVT), early postoperative period, hemoblastosis) were enrolled in the study. We used a case-crossover design to evaluate the sensitivity of new thrombodynamics assay (TD) to the hemostatic state before and after unfractionated heparin (UFH) and low-molecular-weight heparin (LMWH) therapy/prophylaxis and to compare it with the activated partial thromboplastin time (APTT), anti-Xa activity test, thrombin generation test (TGT) and thromboelastography (TEG). A receiver operating characteristic (ROC) curve analysis was used to evaluate changes before and after heparin prophylaxis and therapy. Blood was sampled before heparin injection, at the time of maximal blood heparin concentration and before the next injection.

**Results:**

Hypercoagulation before the start of heparin treatment was detected by TD, TGT and TEG but not by APTT. The area under the ROC curve (AUC) was maximal for TD and anti-Xa, intermediate for TGT and TEG and minimal for APTT.

**Conclusions:**

These results indicate that TD has a high sensitivity to the effects of UFH and LMWH after both prophylactic and therapeutic regimes and may be used for heparin monitoring.

## Introduction

Heparin treatment and prophylaxis for venous thrombosis and thromboembolism (VTE) are widely used in clinical practice. However, wide variability in the effects of unfractionated heparin (UFH) and low-molecular-weight heparin (LMWH) is observed due to different concentrations of platelet factor 4, coagulation factors (antithrombin, fibrinogen and factor VIII) and individual variations in pharmacokinetics [1,2]. Monitoring of LMWH is used in special cases, including patients with extremely low or high body weight, age >75 years, renal disease, critical status or following a change in anticoagulant drug therapy [3–5]. The frequency of bleeding and thrombotic complications during heparin treatment and prophylaxis is 5–10% [6–11]. Therefore, monitoring of heparin efficacy is essential in many clinical situations.

The activated partial prothrombin time (APTT) and anti-Xa activity assays are used in routine control of heparin treatment. APTT is sensitive only to high (therapeutic) doses of UFH [12], and some APTT methods are insensitive to LMWH treatment [13]. The anti-Xa activity assay may be used to monitor both prophylactic and therapeutic doses of UFH or LMWH. However, this assay provides only information about the blood heparin concentration and does not show the overall clotting status of the blood, as the anticoagulant effect of heparin is influenced by individual plasma characteristics (*e*.*g*., concentration of antithrombin and heparin-binding proteins and the ability of the system to generate thrombin). Furthermore, the mechanism of the antithrombotic action of LMWH probably does not only depend upon anti-Xa and anti-IIa activities. For example, UFH and LMWH release Tissue Factor Pathway Inhibitor (TFPI) from vascular sites and this could explain some of the antithrombotic effect of subcutaneously administered LMWH [14]. Anti-Xa level has not been demonstrated to be a good predictor of bleeding risk and antithrombotic efficacy in LMWH thromboprophylaxis and treatment [14]. Additionally, a significant percentage of patients demonstrate resistance to heparin treatment [1,2,15], which cannot be detected using conventional heparin control assays.

Poor efficacy of heparin therapy may also be related to inadequate individual dosing because of insufficiently sensitive methods of laboratory control. Global hemostasis assays superimpose the state of the patient’s clotting system and the effects of anticoagulant treatment. This fact makes global hemostasis assays essential instruments for control of heparin treatment efficacy. Thromboelastography (TEG) and the thrombin generation test (TGT) were shown to be sensitive to the effects of heparin [13,16–23]. Thrombodynamics (TD) is a new global hemostasis assay that was shown to be sensitive to both hypercoagulation and hypocoagulation [24–27]. The aim of our work was to compare the heparin sensitivity of thrombodynamics assay with sensitivity of APTT, TEG and TGT.

## Materials and methods

### Patients and study design

To evaluate assays sensitivity to hypercoagulation we used classical case-control design with use of control group consisting of healthy volunteers (aged 19–65 years) (n = 75). To evaluate assays’ sensitivity to heparin we used a case-crossover design, a variation of a case-control design [28] due to the fact that patients requiring heparin therapy or prophylaxis often have many additional confounding factors (such as presence of concomitant treatment, temporal immobility and baseline hypercoagulation due to the major surgery or malignancy), which makes the selection of appropriate controls rather challenging. To prevent bias from these potential confounders we decided that the best selection would be the cases themselves at the time point before the heparin administration. The number of cases in the area during the study period determined the sample size.

Group 1 consisted of 124 patients with lower extremity DVT (confirmed by ultrasonography and D-dimers level) who were admitted to the Surgical Departments of City Clinical Hospitals #15 and #61 (Moscow, Russia). Subcutaneous injections of UFH (initial dose was 150 IU/kg three times a day, here and below we used IU anti-Xa activity) or LMWH (initial dose was 6,000 IU two times a day) were used for treatment.

Group 2 consisted of 78 early postoperative patients who were admitted to the Surgical Department of the National Research Center for Hematology (Moscow, Russia), the Surgical Department of Altay Department of the National Research Center for Hematology (Barnaul, Russia) and City Clinical Hospital #24 (Moscow, Russia). Patients underwent orthopedic (total hip replacement, 48 patients) and surgical procedures (colon cancer surgery, 10 patients; splenectomy and cholecystectomy, 18 patients; lung biopsy, 2 patients). LMWH prophylaxis by subcutaneous injection (3,000–4,000 IU once a day) was initiated during the first 24 hours after surgery. All patients had VTE Caprini scores of 3 or greater.

Group 3 consisted of 48 patients with hematological malignancies (multiple myeloma, 37 patients; diffuse large B-cell lymphoma, 11 patients) who were admitted to the Chemotherapy Hematologic Diseases Department and the Bone Marrow Transplantation Department of the National Research Center for Hematology (Moscow, Russia). UFH prophylaxis was administered by continuous intravenous infusion (12,000 IU per day depending on the individual risk of VTE). All patients had VTE Caprini scores of 3 or greater.

No thrombotic or bleeding episodes occurred during the investigation.

All the recruitment and analyzing was performed in 2012–2014. The research protocol was approved by the Ethics Committee of the Center for Theoretical Problems of Physicochemical Pharmacology (Protocol #1, September 5, 2011).

The lyophilized human plasma (Hemosil Normal Control Assayed, Instrumentation Laboratory, MA, USA) was used for the in vitro characterization of thrombodynamics. Plasma was prepared according to the manufacturer instructions and was supplemented with human factor XIa (FXIa, 1 pM, Enzyme Research Laboratories, IN, USA) and LMWH (0.16 IU/ml of calcium nodraparin, Aspen Health, France).

To assess the in vitro dose-dependent effect, UFH (5,000 IU/mL, Heparin-Ferein, CJSC Brinsalov-A, Russia) or LMWH (10,000 IU/mL, Enoxaparin sodium, Sanofi Winthrop Industrie, France) were diluted with normal saline and then were added to the whole blood samples of healthy volunteers (150 μL of UFH per 9 mL of citrated blood) to achieve blood concentrations of 0.04, 0.08, 0.17 and 0.33 IU/mL (for UFH) and 0.1, 0.2, 0.3 and 0.4 IU/mL (for LMWH). Normal saline was added to the control samples.

### Blood sampling

Blood samples for coagulation checking were collected at the following thee time points: before the heparin treatment start (Point 0); 2h (for UFH) or 4h (for LMWH) after the heparin injection or 24h after continuous heparin infusion (Point 1, corresponds the maximal heparin concentration [29,30]); and 12h (for group 1) or 24h (for group 2) after heparin injection, immediately before the next injection (Point 2, corresponds the minimal heparin concentration).

Blood was drawn into 10 ml vacuum tubes (Monovette, Sarstedt, Germany) with 106 mM sodium citrate buffer (pH 5.5) at a 9:1 blood:anticoagulant volume ratio through the membrane screw cap needle 21G/8 mm (Multifly, Sarstedt, Germany). The samples were transported to the laboratory within 30 minutes from the blood draw. During transportation, blood samples were stored at 37°C. Whole blood was used for thromboelastography. The remaining blood was processed by centrifugation at 1600g for 15 min to obtain platelet-poor plasma (PPP) and analyzed for APTT and anti-Xa activity assay. A part of PPP was repeatedly processed by centrifugation at 10,000g for 5 min to obtain platelet-free plasma (PFP), which was used for a thrombodynamics assays. The remaining PPP was frozen in liquid nitrogen and stored at −80°C for the thrombin generation assay.

### Laboratory methods

APTT and anti-Xa assay was performed using the Sysmex CA-1500 (Sysmex Corporation, Japan) automated analyzer with Pathromtin SL (Dade Behring, Marburg, Germany) and Berichrom Heparin (Siemens Healthcare Diagnostics, Erlangen, Germany).

TGT was performed in our modification as previously described [24]. Frozen PPP was thawed and incubated in a water bath at 37°C for 1 hour before the experiment. The following reagents were used: 7-amino-4-methylcoumarin (AMC, Sigma Aldrich, St. Louis, MO, USA), phospholipid vesicles prepared by extrusion from phosphatidylcholine and phosphatidylserine (Avanti, Alabaster, AL) at a molar ratio of 7:3 and and stored in a nitrogen atmosphere at 4°C, fluorogenic substrate Z-Gly-Gly-Arg-AMC (Bachem, Bubendorf, Switzerland) and relipidated rabbit tissue factor (Renam, Moscow, Russia). The final concentrations in plasma were: 4 μM of phospholipid; 5 pM of tissue factor and 400 μM of substrate. We used ACTICHROME^®^ TF activity assay (American Diagnostica, GA, USA) for concentration of TF measurement as it was described in [31]. The endogenous thrombin potential (ETP), peak thrombin concentration (Amax), time of peak thrombin concentration (Tmax), and lag time until the appearance of thrombin (LagT) parameters were calculated.

TEG was performed using the TEG 5000 Hemostasis Analyzer System (Haemonetics Corporation, USA) and reagents. For Ca^2+^ level restoration and to start clotting, 0.2 M calcium chloride solution was used. The reaction time (R), clot formation time (k), alpha angle (α) and maximal amplitude (MA) parameters were calculated.

**Fig 1:**
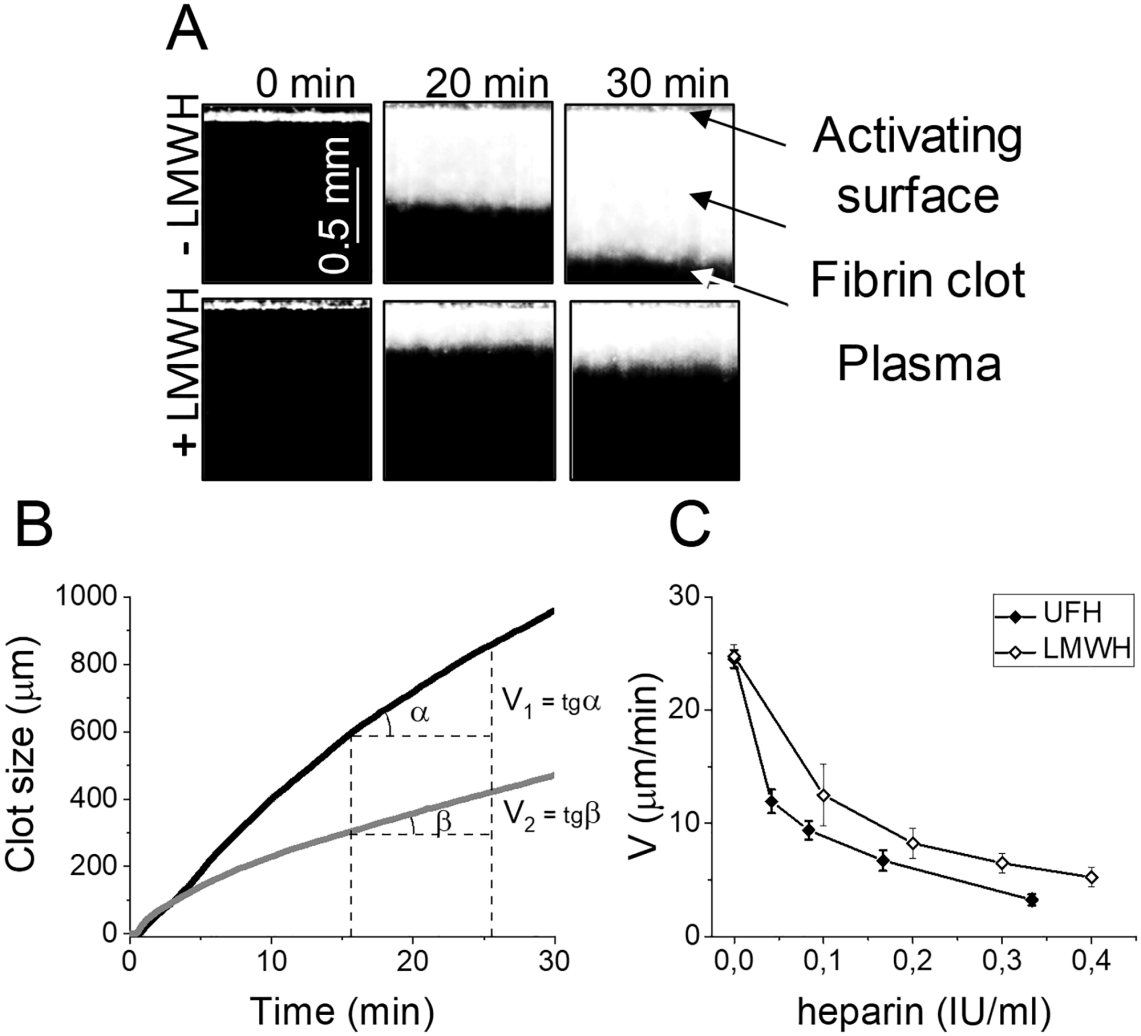
The thrombodynamics method principle. (A) The photos of fibrin clot growth are shown for normal plasma and in LMWH presence (0.08 IU/ml). The edge of the activator on the top of the pictures is covered with immobilized tissue factor. Clot starts growing from the edge of the activator to the bulk of the plasma. The process of fibrin clot formation is recorded in a time-lapse video microscopy mode by means of dark-field light scattering method. The obtained series of photos shows how the size of fibrin clot changes over time. (B) Plot of clot size versus time in normal plasma (black line) and in LMWH presence (0.1 IU/ml) representing the principle of the parameter V calculation. (C) The in vitro heparin dose dependency for V in the presence of UFH or LMWH in plasma of healthy volunteers. Means and SEM were shown (n = 10).

TD was performed using a Thrombodynamics Analyzer and Thrombodynamics kit (LLC HemaCore, Moscow, Russia) consisting of corn trypsin inhibitor (CTI), calcium acetate and a plastic insert with immobilized relipidated recombinant TF from Instrumentation Laboratory (Bedford, MA, USA). Manufacturer (LLC Hemacore) provided the information about density of the TF in the Thrombodynamics kits: 113±26 pmoles/m^2^; ACTICHROME^®^ TF activity assay was used. Plasma preparation, experimentation and image processing were performed as previously described [24]. This method is based on registering spatial fibrin clot growth after activation of clotting in a thin layer of plasma after contact with an immobilized tissue factor bearing surface. The process of clot growth was registered by serial photography during the test (Fig 1A). Based on the photos, a plot of clot growth versus time was obtained (Fig 1B). The lag time (Tlag, the delay between the test start and the onset of clot formation), the initial velocity of clot growth (Vi, calculated as the mean clot growth velocity over a Tlag+2 to Tlag+6 interval), the stationary velocity of clot growth (Vst, calculated as the mean clot growth velocity over a Tlag+15 min to Tlag+25 min interval), the velocity of clot growth (V, the parameter, calculated as the mean clot growth velocity over the 10-min interval before spontaneous clotting occurs and equal to Vst in cases without spontaneous clot formation) were calculated.

Reference values were determined by testing plasma obtained from healthy volunteers (aged 19–65 years): n = 75, 33, 25 and 75 for APTT, TGT, TEG and TD, respectively.

### Statistical analysis

Statistical analysis was performed using Origin Pro 8 (Origin-Lab Corp., USA) and MedCalc (Belgium) software. The mean value, median value, standard deviation (SD) and coefficient of variation (CV) were used to estimate the assay results. The nonparametric pair sample Wilcoxon signed rank test and Mann-Whitney U-test were used for analysis, the significance level was set at P<0.05.

The relative effect (Q) of FXIa or FXIa+LMWH on parameters Vi, Vst, V, CS and D in vitro was calculated as:

$$Q=modul(\frac{P_{n}-P_{mod}}{{SD}_{n}-{SD}_{mod}})$$

where P_*n*_ and SD_*n*_—mean value and standard deviation of parameter in normal plasma and P_*mod*_ and SD_*mod*_—mean value and standard deviation of parameter in plasma supplemented with FXIa or FXIa+LMWH. Q was reversed for Tlag.

To estimate the coagulation tests’ sensitivity during heparin therapy a receiving operating curve (ROC) curve analysis was used. The presence or absence of anticoagulant therapy was selected as an “outcome”. A standard binomial exact statistics and Bootstrap estimation (analysis of 3000 alternative ROC-curves) were used for the calculation of the parameters: area under the curve (AUC) and cut-off value for each parameter. We considered the sensitivity as low when AUC was less than 0.7, moderate when AUC was from 0.7 to 0.8 and high when AUC was more than 0.8. We compared the sensitivity to heparin of all the parameters within each assay and presented data of only one parameter from each assay with the better sensitivity. After obtaining the cut-off, sensitivity and specificity, positive and negative predictive values of each test parameter were calculated.

### Potential sources of bias

The main potential source of bias is our inability to measure all tests in all of the participants. To deal with missing data we restricted analyses to individuals with complete data on all variables required for a particular analysis.

## Results

### Thrombodynamics variability and sensitivity in the in vitro system

We used the lyophilized human plasma to characterize TD parameters. FXIa was added to imitate the hypercoagulant plasma state [32]. LMWH (0.16 IU/ml) was additionally added to imitate the heparin prophylaxis effect. The characteristic parameters were presented in S1 Table. Clot growth velocity (V) showed the highest relative effect (Q) of the factor XIa and FXIa+LMWH so in the following sections of the paper we demonstrated only this parameter of TD.

We examined in vitro dose-dependent effect of UFH and LMWH on V in TD (Fig 1C). Heparin concentration in blood was varied within the range of 0–0.33 IU/ml for UFH and 0–0.4 IU/ml for LMWH that approximately corresponded to plasma concentration of 0–0.57 IU/ml and 0–0.69 IU/ml, respectively (based on mean hematocrit value of 42%). These heparin concentrations are characteristic for treated patients [29,30].

### Thrombodynamics vs APTT

We included patients from three high-VTE-risk groups: 1) DVT, 2) early postoperative period and 3) hemoblastosis. A total of 296 participants were enrolled in the study; 250 patients were included in the analysis, and 46 were excluded because a blood sample was not collected at Point 0. Patients’ characteristics are shown in Table 1.

**Table 1:** Characteristics of patients.

Group	n	m/f	Age, years median (min-max)	Heparin type	Heparin dosage	Point 0/Point 1/Point 2					
						Time*	APTT n	TD n	Anti-Xa n	TGT n	TEG n
1 DVT	24	14/10	59 (24–86)	LMWH	6000 IU 2x a day	Bef/3h/12h	24/21/23	23/21/22	24/21/24	-	-
	71	46/25	57 (21–84)	UFH	150 IU/kg 3x a day	Bef/2h/8h	64/67/64	64/58/64	-	54/50/50	-
	29	15/14	67 (28–87)	UFH	150 IU/kg 3x a day	Bef/-/8h	29/-/25	29/-/28	-	-	-
2 SURGERY	42	21/21	60 (37–76)	LMWH	4000 IU 1x a day	Bef/-/24h	29/-/39	13/-/22	-	18/-/32	27/-/38
	26	11/15	51 (20–75)	LMWH	3000 IU 1x a day	Bef/3h/24h	26/25/26	26/26/26	24/26/25	10/10/16	24/23/22
	10	3/7	54 (26–65)	LMWH	4000 IU 1x a day	Bef/3h/24h	5/10/9	5/10/9	5/10/9	-	-
3 ONCO	34	21/13	56 (27–65)	UFH	1200 IU/d	Bef/24h^†^/NA	30/27/ NA	30/27/ NA	-	27/23/ NA	29/27/ NA
	14	4/10	43 (23–80)	UFH	1200 IU/d	Bef/24h^†^/NA	14/14/ NA	14/14/ NA	-	-	13/12/ NA

DVT—patients with lower extremity DVT; SURGERY—early postoperative patients; ONCO—patients with hematological malignancies; APTT—activated partial thromboplastin time; TD—thrombodynamics; TGT—thrombin generation test; TEG—thromboelastography; m/f = male/female; Bef—before administration of heparin; NA—not applicable; n—number of participants.

* Time between heparin injection and blood sampling (at maximal blood heparin concentration).

^†^ 24h after continuous heparin infusion.

**Fig 2:**
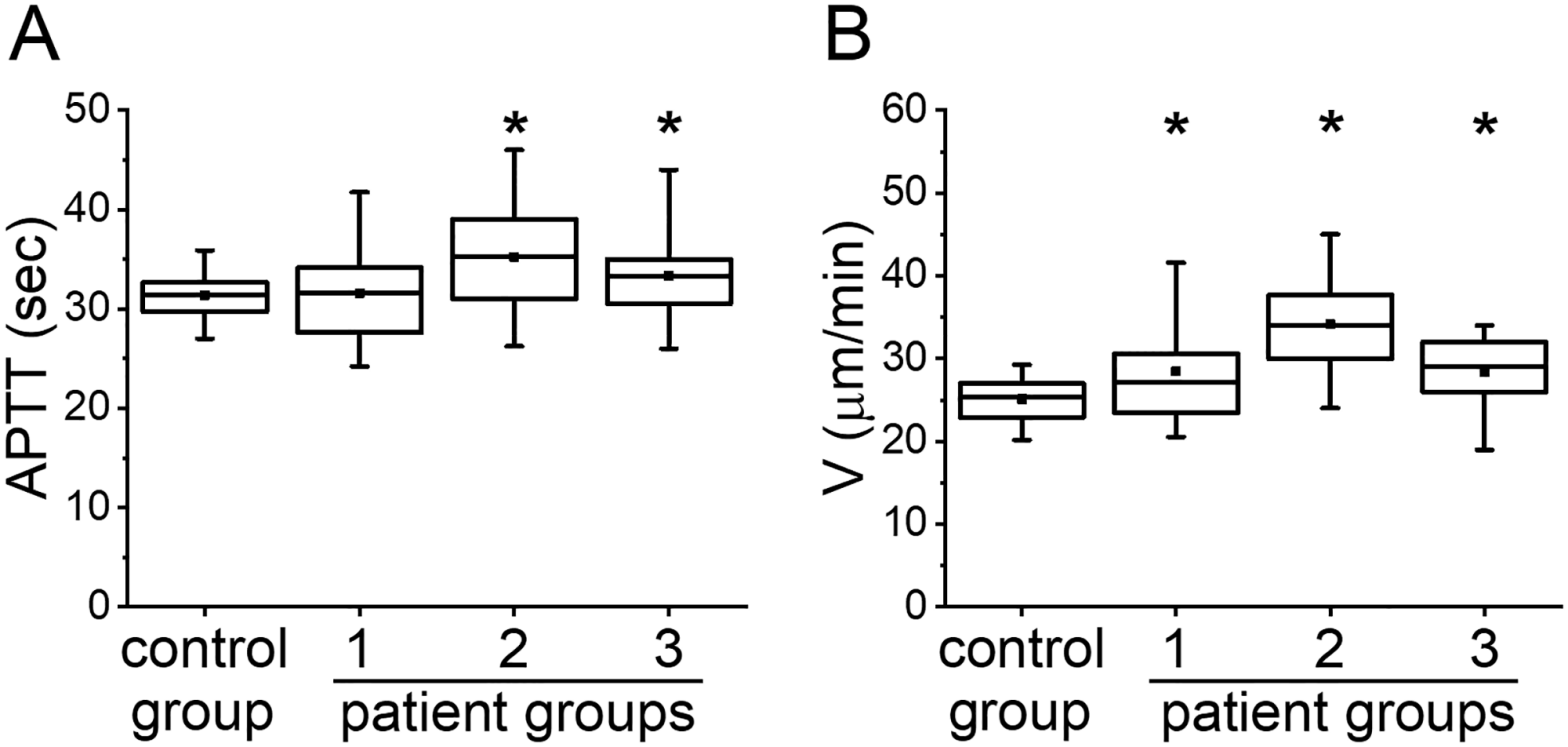
APTT vs TD parameters before heparin treatment. (A) APTT and (B) V in TD before heparin treatment in groups: healthy volunteers (control group), group 1, group 2 and group 3. The box plots indicate the following parameters: the mean value (the dot inside the box), the median (the horizontal line inside the box), the 25th and 75th percentiles (the bottom and top of the box, respectively) and the 5th and 95th percentiles (the ends of the whiskers). * indicates a significant difference from healthy volunteers group (p<0.01, Mann-Whitney test).

To characterize the initial state of patients before anticoagulation (Point 0), we compared the results of APTT and TD of patients with those of healthy volunteers (Fig 2). We only presented data for V in TD because it turned out to be the most sensitive to heparin treatment.

The number of patients in each group for which APTT was measured is reported in Table 1. APTT was not significantly different from healthy volunteers in group 1 and was prolonged in groups 2 and 3. Parameter V in TD was increased in all three groups of patients thus demonstrated hypercoagulant state. Therefore, the parameter V was able to detect hypercoagulation for patients in all high VTE risk groups while APTT remained in the normal range or even prolonged, which is generally interpreted as hypocoagulation.

Changes in APTT and V after heparin treatment were presented in S2 Table. APTT was significantly prolonged only for the UFH treatment while V was significantly decreased for both UFH and LMWH treatment.

**Fig 3:**
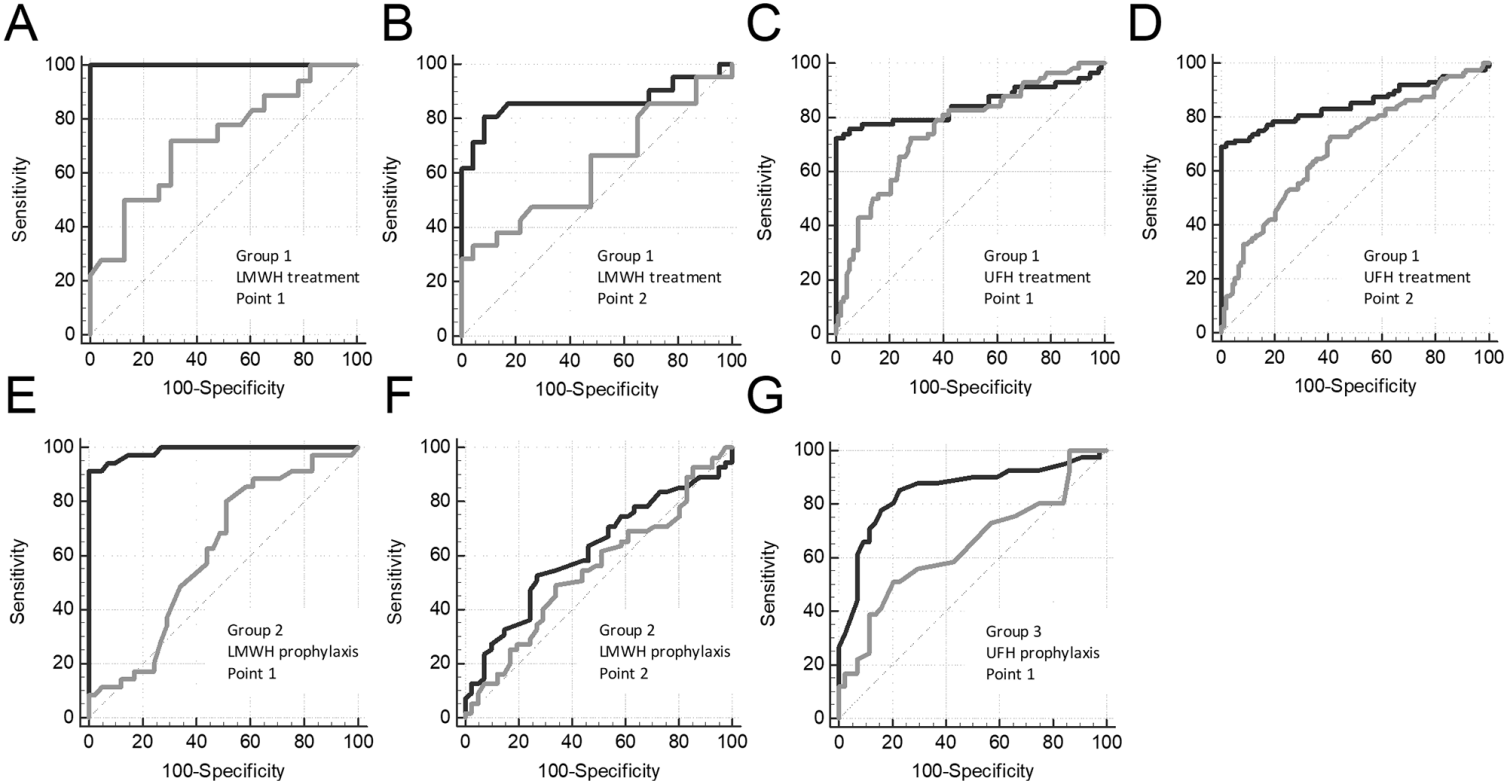
Heparin effect on APTT vs TD. ROC curves for APTT (gray lines) and V in TD (black lines) for treatment with LMWH (group 1) at the Point 1 (A) and Point 2 (B) or UFH (group 1) at the Point 1 (C) and Point 2 (D) and prophylactics with LMWH (group 2) at the Point 1 (E) and Point 2 (F) or UFH (group 3) at Point 1 (G). Data of the same patients before the first heparin injection were used as controls.

We compared the sensitivity of APTT and V to heparin treatment using ROC analysis. The data set of patient’s samples before treatment was termed “controls” (there was no heparin in the blood samples), and the data set of patient’s samples after treatment was termed “cases” (there was heparin in the blood samples). To perform this analysis, we used only samples with both APTT and TD data present. We performed ROC analysis separately for Point 1 and Point 2 to obtain data about heparin effect at maximal and minimal blood concentration, respectively. The ROC curves for the APTT and V are presented in Fig 3, and the parameters of ROC-analysis are shown in S3 Table. APTT was moderately sensitive to LMWH therapy in group 1 at Point 1 (AUC = 0.720) and insensitive at Point 2 (AUC = 0.623; not significantly different from the 0.5 level) whereas V had a high sensitivity at both Point 1 and Point 2 (AUC = 1.000 and 0.864; significantly higher compared with the AUC for APTT, P<0.05). APTT was moderately sensitive to UFH therapy in group 1 at Point 1 (AUC = 0.755) and had a low sensitivity at Point 2 (AUC = 0.683) whereas V had a high sensitivity at both Point 1 (AUC = 0.849, P>0.05) and Point 2 (AUC = 0.850; significantly higher compared with the AUC for APTT, P<0.05). APTT was insensitive to LMWH prophylaxis in group 2 at both Point 1 and Point 2 (AUC = 0.611 and 0.545; not significantly different from the 0.5 level) whereas V had a high sensitivity at Point 1 (AUC = 0.897; significantly higher compared with the AUC for APTT, P<0.05) and had a low sensitivity at Point 2 (AUC = 0.616, P = 0.42). APTT had a low sensitivity to UFH prophylaxis in group 3 at Point 1 (AUC = 0.644), V had a high sensitivity at Point 1 (AUC = 0.851; significantly higher compared with the AUC for APTT, P<0.05).

### Thrombodynamics vs anti-Xa assay

**Fig 4:**
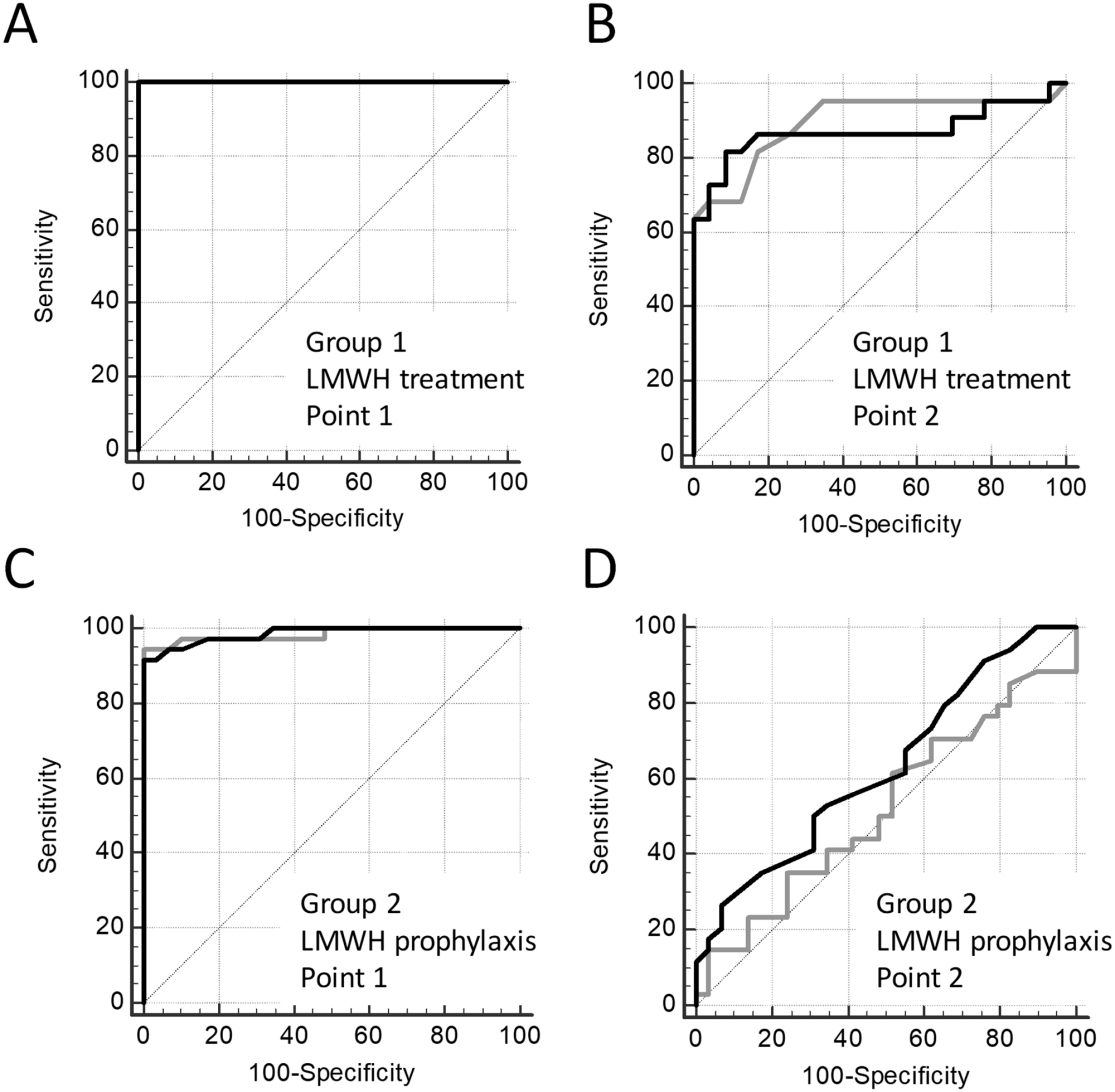
Heparin effect on anti-Xa vs TD. ROC curves for anti-Xa (gray lines) and V in TD (black lines) for treatment with LMWH (group 1) at Point 1 (A) and Point 2 (B) and prophylaxis with LMWH (group 2) at Point 1 (C) and Point 2 (D). Data of the same patients before the first heparin injection were used as controls.

The number of patients in each group for which anti-Xa was measured is reported in Table 1. Anti-Xa was measured only in LMWH therapy and prophylaxis groups, so the comparison of anti-Xa and TD was performed only in these patients. The ROC curves for the anti-Xa and V are presented in Fig 4, and the parameters of ROC-analysis are shown in S4 Table. To perform this analysis, we used only samples with both anti-Xa and TD data present.

Both assays demonstrated equal sensitivity to treatment with therapeutic and prophylactic LMWH doses. Anti-Xa assay had a high sensitivity to LMWH therapy both at Point 1 (AUC = 1.000) and at Point 2 (AUC = 0.896), V in TD showed same results both at Point 1 (AUC = 1.000) and at Point 2 (AUC = 0.871). For LMWH prophylaxis anti-Xa assay had a high sensitivity at Point 1 (AUC = 0.984) but low sensitivity at Point 2 (AUC = 0.522; not significantly different from 0.5 level). V in TD showed the same tendency both at Point 1 (AUC = 0.986) and at Point 2 (AUC = 0.629; not significantly different from 0.5 level). No significant differences between AUC of anti-Xa and V was shown in any of the treatment regimes.

### Thrombodynamics vs thrombin generation test

**Fig 5:**
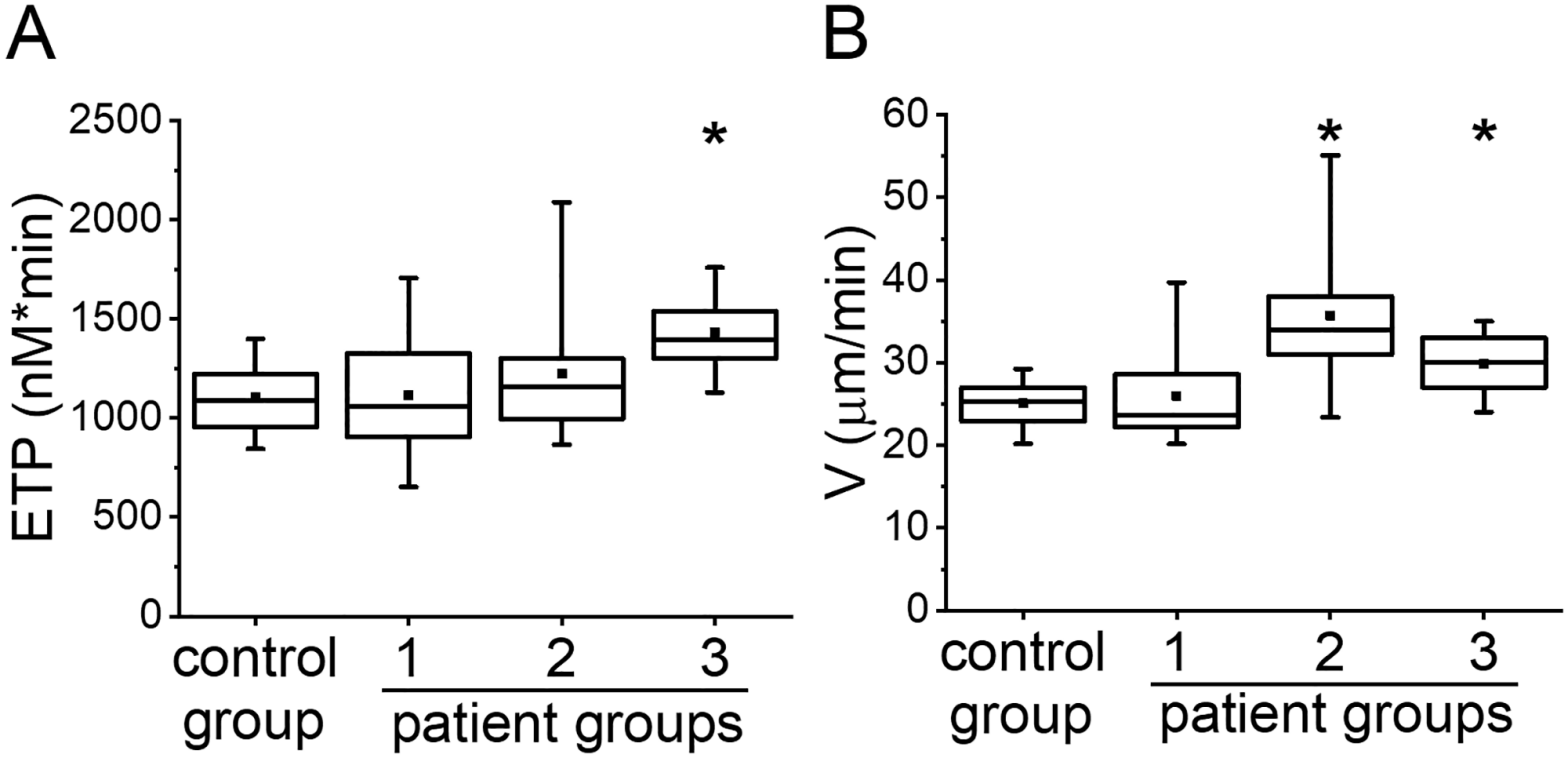
TGT vs TD before heparin treatment. (A) ETP in TGT and (B) V in TD before heparin treatment in groups: healthy volunteers (control group), group 1, group 2) and group 3. The box plots indicate the following parameters: the mean value (the dot inside the box), the median (the horizontal line inside the box), the 25th and 75th percentiles (the bottom and top of the box, respectively) and the 5th and 95th percentiles (the ends of the whiskers). * indicates a significant difference from healthy volunteers group (p<0.01, Mann-Whitney test).

Number of patients in each group for which TGT was measured is reported in Table 1. To characterize the initial state of patients before anticoagulation, we compared the results of TGT and TD of patients with those of healthy volunteers (Fig 5). We only presented data for ETP in TGT and V in TD because these parameters turned out to be the most sensitive to heparin treatment.

ETP in TGT was significantly higher than in healthy volunteers in group 3 and was not significantly different from healthy volunteers in groups 1 and 2. V in TD was increased in groups 2 and 3 but was not different from healthy volunteers in group 1. The difference in results demonstrated in Figs 2B and 5B was due to decrease the patient’s number for correct comparison with TGT. Both TGT and TD were able to detect higher VTE risk for patients in group 3.

**Fig 6:**
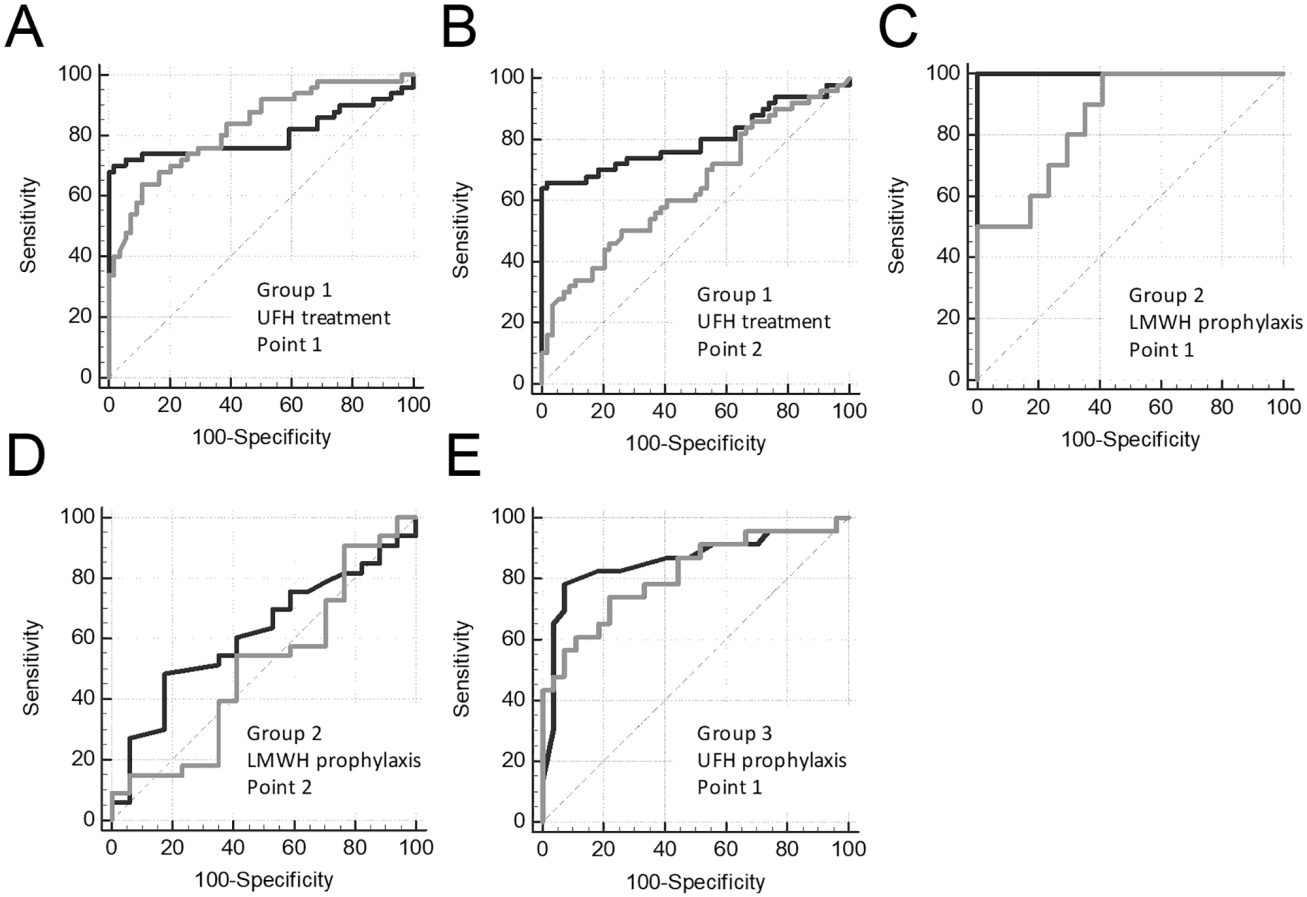
Heparin effect on TGT vs TD. ROC curves for ETP in TGT (gray lines) and V in TD (black lines) for treatment with UFH (group 1) at Point 1 (A) and Point 2 (B) and prophylaxis with LMWH (group 2) at Point 1 (C) and Point 2 (D) or UFH (group 3) at Point 1 (E). Data of the same patients before the first heparin injection were used as controls.

The ROC curves for the ETP and V are presented in Fig 6, and the parameters of ROC-analysis are shown in S5 Table. To perform this analysis, we used only samples with both TGT and TD data present. ETP had a high sensitivity to UFH therapy in group 1 at Point 1 (AUC = 0.826) and low sensitivity at Point 2 (AUC = 0.641) whereas V had a high sensitivity at both Point 1 (AUC = 0.802) and Point 2 (AUC = 0.800; significantly higher compared with the AUC for ETP, P<0.05). ETP had a high sensitivity to LMWH prophylaxis in group 2 at Point 1 (AUC = 0.853) and low sensitivity at Point 2 (AUC = 0.504; not significantly different from the 0.5 level), V had a high sensitivity at Point 1 (AUC = 1.00; significantly higher compared with the AUC for ETP, P<0.05) and low sensitivity at Point 2 (AUC = 0.616; not significantly different from the 0.5 level). ETP had a high sensitivity to UFH prophylaxis in group 3 at Point 1 (AUC = 0.813), V had a high sensitivity too (AUC = 0.859).

### Thrombodynamics vs thromboelastography

**Fig 7:**
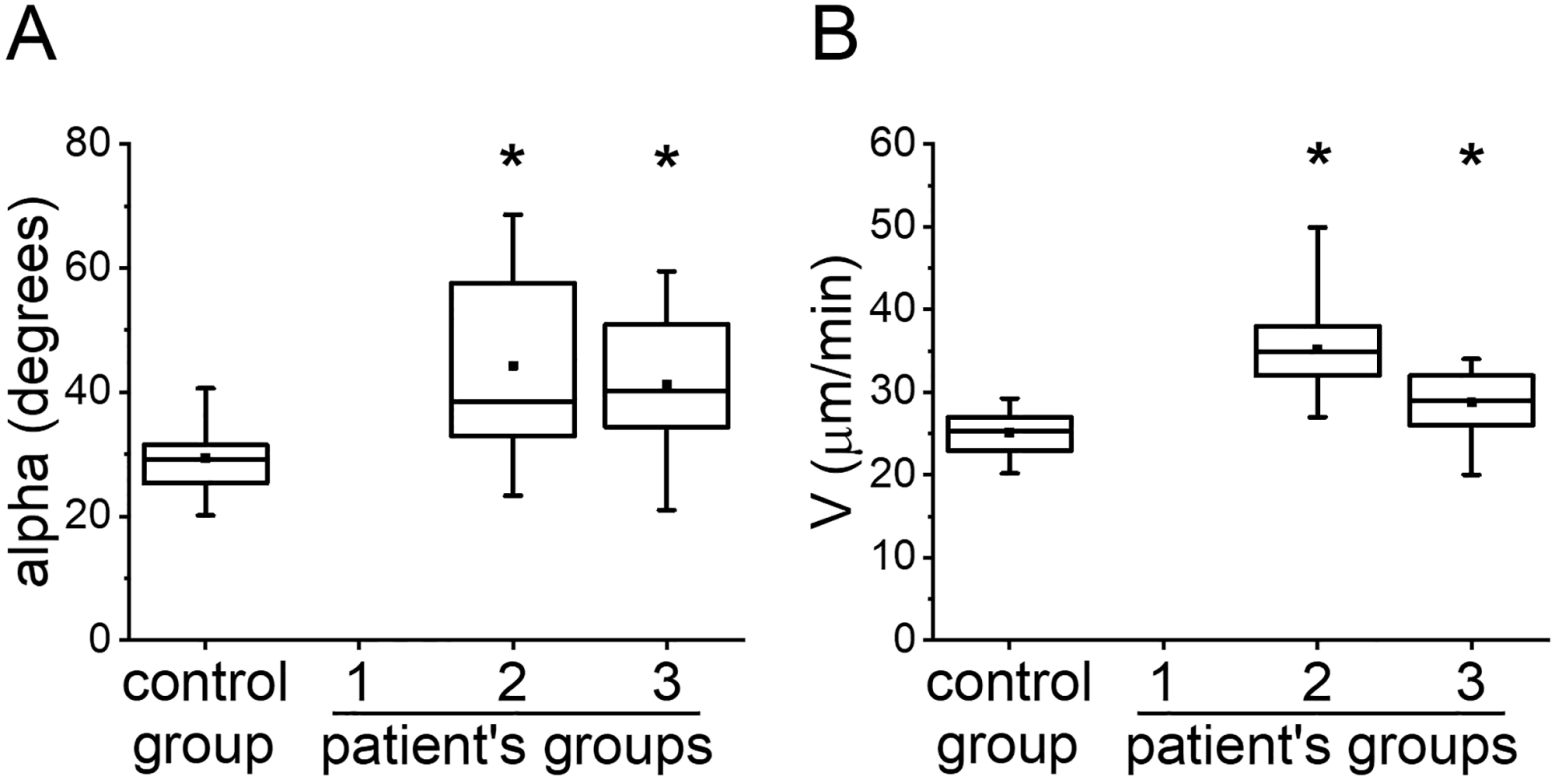
TEG vs TD before heparin treatment. (A) alpha in TEG and (B) V in TD before heparin treatment in groups: healthy volunteers (control group), group 2 and group 3. The box plots indicate the following parameters: the mean value (the dot inside the box), the median (the horizontal line inside the box), the 25th and 75th percentiles (the bottom and top of the box, respectively) and the 5th and 95th percentiles (the ends of the whiskers). * indicates a significant difference from healthy volunteers group (p<0.01, Mann-Whitney test).

Number of patients in each group for which TEG was measured is reported in Table 1. To characterize the patients’ state before anticoagulation, we compared the results of TEG and TD of patients with those of healthy volunteers (Fig 7). We only presented data for alpha in TEG and V in TD because it turned out to be the most sensitive to heparin treatment.

Alpha in TEG in groups 2 and 3 was significantly higher than in healthy volunteers as well as V in TD. We decreased the patient’s number in TD for correct comparison with TEG. Both TEG and TD were able to detect high VTE risk for patients in groups 2 and 3.

**Fig 8:**
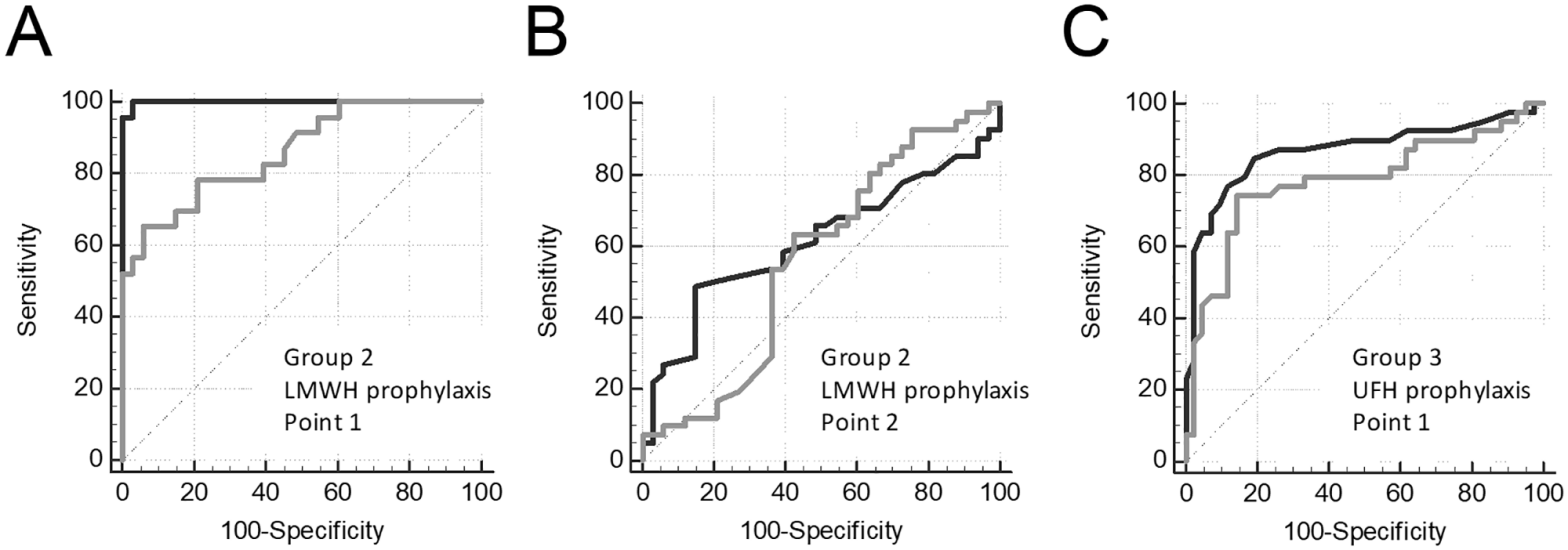
Heparin effect on TEG vs TD. ROC curves for alpha in TEG (gray lines) and V in TD (black lines) for prophylaxis with LMWH (group 2) at Point 1 (A) and Point 2 (B) or UFH (group 3) at Point 1 (C). Data of the same patients before the first heparin injection were used as controls.

The ROC curves for the angle alpha and V are presented in Fig 8, and the parameters of ROC-analysis are shown in S6 Table. To perform this analysis, we used only samples with both TEG and TD data present. Alpha had a high sensitivity to LMWH prophylaxis in group 2 at Point 1 (AUC = 0.861) and low sensitivity at Point 2 (AUC = 0.561; not significantly different from the 0.5 level), V had a high sensitivity at Point 1 (AUC = 0.999; significantly higher compared with the AUC for alpha, P<0.05) and low sensitivity at Point 2 (AUC = 0.613; not significantly different from the 0.5 level). Alpha was moderately sensitive to UFH prophylaxis in group 3 at Point 1 (AUC = 0.777), V had a high sensitivity at Point 1 (AUC = 0.864). Therefore, sensitivity to heparin treatment of TD was at least comparable to sensitivity of TEG.

## Discussion

Thrombodynamics assay is a new global hemostasis assay; the first publications on this technology date back to the early 2000s [33–35]. Heparins effect on thrombodynamics was published for small cohorts of patients or in case-reports [24,25,27]. The earlier study presenting the comparison of thrombodynamics with other standard and global coagulation assays using in vitro setting has already shown that thrombodynamics is a stable, reproducible and sensitive test with narrow parameter distributions which accurately describes pharmacodynamics of any heparin [36]. To confirm the utility of this method for clinical use, we studied the effects of heparin for the APTT, TGT, TEG and TD assays ex vivo in blood samples of patients undergoing heparin treatment. We evaluated both UFH and LMWH effects in therapeutic and prophylactic regimes of treatment.

The intra-laboratory variability of TD’s parameters was 2.4% for the clot growth velocity V, which is comparable with the APTT‘s variability (about 2%, [37]) and better than variability of TGT (13% for ETP [38]) and TEG (10% for alpha [39]). V was the most sensitive to heparin effect compared with the other TD parameters; V continuously decreased with increase of heparin concentration within all range of possible heparin concentrations in the patients’ blood. The UFH effect on V was more pronounced compared with LMWH. The reason may be that we built the dependence based only on their anti-Xa activity, whereas UFH also has equal anti-Xa and anti-thrombin activity [40].

We assessed APTT, TEG, TGT and TD to reveal the hypercoagulation in patients at high risk of VTE. APTT did not reflect changes in any of the patient groups; moreover, APTT was prolonged in groups 2 and 3 that can be wrongly regarded as an increased risk of bleeding. TD demonstrated significant hypercoagulant changes in all patients groups or at least in groups 2 and 3, TGT–only in group 3, TEG–in groups 2 and 3 (for group 1 TEG was not measured).

APTT prolongation in groups 2 and 3 could be due to decrease of coagulation factors concentration after blood loss/blood dilution during surgery or coagulation factors consumption. This is indirectly confirmed by our previous study. We have already observed prolonged APTT (38±7 sec while normal range was 25.1–36.6 sec) and normal to hypercoagulant V in TD (V>20 μm/min) in patients receiving warfarin therapy after cardiac surgery (S1 Fig) [41]. Concentrations of vitamin-K dependent clotting factors were significantly reduced compared with the normal value (100%): 32±14% for prothrombin, 22±14% for factor VII, 69±27% for factor IX and 24±15% for factor X.

We speculate that TEG, TGT and TD reflected the real coagulation state of these patients. Thus, the hypercoagulation observed in these assays may be a sign of the higher VTE risk, while the normalization or even shift to hypocoagulation during effective heparin therapy may be a sign of decreased VTE risk. Surprisingly, TGT showed the lowest hypercoagulation sensitivity among global assays tested. The lack of sensitivity of TGT to hypercoagulation preceding heparin injection in some of the groups can be, at least partially, explained by insufficient sample size. To deal with missing data we restricted analyses to individuals with complete data on all variables required for a particular analysis (as stated in Materials and methods section). However, in thrombodynamics even after reduction of the sample, required for correct comparison with TGT, all the tendencies remained visible and significant, except for the difference between control group and group 1. So, if there were any evidence that TGT registers hypercoagulation in groups 1 and 2, we expected to observe it even on this sample size.

In order to perform the comparison of the sensitivity of tests to heparins we have identify three grades of sensitivity: low (AUC<0.7), medium (AUC Є [0.7–0.8]) and high (AUC>0.8). The gradations for all time points, heparin type and dosages for each assay are presented in S7 Table. For each assay, we determined the proportion of situations where TD have a better sensitivity to heparin treatment. We found that TD is more sensitive than APTT in 100% of cases (n = 7), it is comparable to anti-Xa in 100% of cases (n = 4), it is more sensitive than Amax in TGT in 60% of cases (n = 5) and it is more sensitive than alpha in TEG in 67% of cases (n = 3). There were no cases when TD had a sensitivity to heparin lower than any other assay. All assays had a low sensitivity to heparin at Point 2 during prophylactic treatment. Thus, TD showed a better heparin sensitivity than APTT, comparable to Anti-Xa and better or comparable to TGT and TEG.

Separately, we would like to discuss the mechanisms by which thrombodynamics is so highly sensitive to heparins. Heparin is a cofactor of antithrombin and enhances its affinity for clotting factors: thrombin, Xa and IXa. The thrombodynamics assay allows us to identify the phase of the propagation, for which the complexes of internal tenase and protrombinase are responsible [42]. Both complexes contain factors that are inhibited by antithrombin: IXa and Xa, respectively. The activation phase, localized in the thrombodynamics assay at the surface with tissue factor, includes external tenase and prothrombinase. Antithrombin does not affect the external tenase. Thus, it turns out that the effect of heparin is more pronounced in the phase of propagation. This is consistent with the data obtained in our work and a comparison of the sensitivity of thrombodynamics to heparin in vitro [36]. Thus, the clot growth velocity is most sensitive to the effect of heparin.

Since we used related data we performed an additional calculation using the Bootstrap estimation [43]. We did not find any significant difference from the parameters calculated via binomial exact method (S8 Table).

Very different individual patients’ responses to heparin treatment were detected with the global hemostasis assays. We found that up to 21% of patients were in a state of hypercoagulation during heparin treatment at Point 1, while 5–70% of patients were in a state of hypercoagulation at Point 2 (S9 Table). This finding may indicate the inefficiency of heparin treatment and the higher thrombotic risk for these patients. This is an area for further clinical studies.

The main limitation of our study is that we measured anti-Xa assay, TGT and TEG only for a part of patients, so we obtained small groups and relatively large variability. Thus, TEG was not performed for patients on heparin therapy; TGT was not performed for patients on LMWH therapy. This reduces our ability to compare the sensitivity of the tests in these cases. However, comparison of the sensitivity of these assays with TD was performed only for paired data, so the comparison itself is objective and trustworthy.

In our research we used only one APTT reagent for comparison with TD. Various APTT reagents do have different sensitivity to heparin. However, from the literature we know that not only the reagent type do influence APTT sensitivity to heparins, but also the model of coagulometer does influence it [44]. For most of the reagents mentioned in literature the correlation of APTT with anti-Xa activity results varies in the range of r = 0.7–0.8 [44,45]. We decided not to focus on comparison of various APTT reagents for the sake of standardization of protocol in all clinical settings of various specialty, which took part in the study. We chose Pathromtin SL mainly for two reasons: 1) it is widely used; 2) it may be not the most heparin-responsive reagent, but it cannot also be considered low-responsive (as are, for example, Actin or IL Test [44]). We do hope that this coagulometer-reagent pairing (Sysmex CA-1500+Pathromtin SL) may, to some extent, represent a kind of "average" APTT, enough to illustrate, at least, qualitative difference between the assays mentioned in the article.

We compared assays that used different sample preparation (regarding either whole blood in TEG or platelet free plasma in TD). The decreased heparin effect in TEG may be due to the platelet factor 4 from alpha granules binding to heparin molecules [46]. However, our task was to compare clinical efficacy of TEG and TD, so we consider our comparison to be correct.

Our study showed that the thrombodynamics assay is effective for monitoring heparin. TD is more sensitive to heparin than APTT and, apparently, its sensitivity is comparable to anti-Xa sensitivity and comparable or higher than that of TGT and TEG. Further clinical studies are needed to determine whether thrombodynamics can detect cases of ineffective therapy leading to thrombosis or bleeding.

## Supporting information

S1 Table Intra-laboratory variation of thrombodynamics. (DOCX)

S2 Table Heparin effect on APTT and V in TD. (DOCX)

S3 Table Heparin sensitivity: APTT and V in TD. (DOCX)

S4 Table Heparin sensitivity: Anti-Xa activity and V in TD. (DOCX)

S5 Table Heparin sensitivity: ETP in TGT and V in TD. (DOCX)

S6 Table Heparin sensitivity: Alpha in TEG and V in TD. (DOCX)

S7 Table Comparison the sensitivity of hemostasis assays to heparins. (DOCX)

S8 Table ROC analysis with Bootstrap estimation (3000 random curves): APTT vs V in TD. (DOCX)

S9 Table Heparin treatment effects on hemostasis assays. (DOCX)

S1 Fig Correlation between APTT and V in Thrombodinamics. Data for 32 patients receiving warfarin therapy (5 mg/day) on 3–5 day after cardiac surgery. Reorganized data from Goncharova et al, 2015.
(TIF)
