# Supplementary material for: Thrombodynamics—A new global hemostasis assay for heparin monitoring in patients under the anticoagulant treatment
Source: PLoS One. 2018 Jun 28;13(6):e0199900. doi: 10.1371/journal.pone.0199900 (PMC6023127; doi:10.1371/journal.pone.0199900)
Supplement: S1 Table — (DOCX) [file pone.0199900.s001.docx]

**S1 Table.** **Intra-laboratory variation of thrombodynamics**

| **Parameter** | **Units** | **Normal plasma** | **Hypercoagulant plasma** | **Heparin effect on hypercoagulant plasma** |
| --- | --- | --- | --- | --- |
| Tlag (min) | min | 0.79 (11.2) | 0.90 (14.5), Q=0.5 | 0.91 (13.3), Q=0.6 |
| Vi (µm/min) | µm/min | 66.5 (2.4) | 58.1 (2.4), Q=2.8 | 42.5 (3.4), Q=7.9 |
| Vst (µm/min) | µm/min | 33.9 (2.4) | ND | 14.7 (5.2), Q=12.2 |
| V (µm/min) | µm/min | 33.9 (2.4) | 56.2 (7.9), Q=4.2 | 14.7 (5.2), Q=12.2 |
| CS (µm) | µm | 1431 (2.0) | ND | 806 (3.6), Q=10.7 |
| D (a.u.) | a.u. | 22376 (10.0) | 19799 (9.5), Q=0.6 | 19988 (9.0), Q=0.6 |
| Tsp (min) | min | NS | 17.9 (14.0) | NSp |

Mean (standard variation in %) and Q are presented for n=16 repeats. A.u. – arbitrary units of light scattering intensity; ND – parameter can not be determined due to spontaneous clotting; NSp – no spontaneous clotting during 45 minutes was registered.
