# Supplementary material for: Thrombodynamics—A new global hemostasis assay for heparin monitoring in patients under the anticoagulant treatment
Source: PLoS One. 2018 Jun 28;13(6):e0199900. doi: 10.1371/journal.pone.0199900 (PMC6023127; doi:10.1371/journal.pone.0199900)
Supplement: S2 Table — (DOCX) [file pone.0199900.s002.docx]

**S2 Table. Heparin effect on APTT and V in TD**

| **Group** | **1** | | **2** | **3** |
| --- | --- | --- | --- | --- |
| Heparin type | UFH | LMWH | LMWH | UFH |
| Before treatment:  APTT (sec)  V (mm/min) | 31.8±9.1^*^  27.8±7.4 | 31.0±4.1  31.2±4.7 | 36.9±7.5  35.1±8.2 | 33.3±5.2  28.1±10.4 |
| Point 1:  APTT (sec)  V (mm/min) | 39.1±14.1  16.2±11.6 | 33.4±4.4  10.5±4.8 | 38.0±8.6  15.5±5.5 | 38.2±10.3  17.5±7.5 |
| P^†^:  APTT  V | **<0.001**  **<0.001** | 0.052  **<0.001** | 0.494  **0.006** | **0.001**  **<0.001** |
| Point 2:  APTT (sec)  V (mm/min) | 39.7±21.8  16.8±10.3 | 33.2±5.3  22.5±7.8 | 38.1±18.2  32.3±10.0 | -  - |
| P^†^:  APTT  V | **<0.001**  **<0.001** | 0.059  **<0.001** | 0.990  **0.038** | -  - |

APTT – activated partial thromboplastin time; TD – thrombodynamics; UFH – unfractionated heparin; LMWH – low molecular weight heparin

^*^ Mean values ± SD were presented.

^†^ Paired Sample Wilcoxon Single Rang test was used.
