# Supplementary material for: Thrombodynamics—A new global hemostasis assay for heparin monitoring in patients under the anticoagulant treatment
Source: PLoS One. 2018 Jun 28;13(6):e0199900. doi: 10.1371/journal.pone.0199900 (PMC6023127; doi:10.1371/journal.pone.0199900)
Supplement: S3 Table — (DOCX) [file pone.0199900.s003.docx]

**S3 Table. Heparin sensitivity: APTT and V in TD**

| **Group** | **Heparin type** | **Heparin dosage** | **Point #** | **Test** | **Positive group, n** | **Negative group, n** | **AUC** | **95% CI** | **P (AUC>0.5)** | **Cut-Off** | **Sensitivity** | **Specificity** | **Positive predictive value** | **Negative predictive value** | **P** |
| --- | --- | --- | --- | --- | --- | --- | --- | --- | --- | --- | --- | --- | --- | --- | --- |
| 1 | LMWH | 6000 IU 2x a day | 1 | APTT | 18 | 23 | 0.720 | 0.558-0.849 | 0.0073 | >32.8 | 72.2 | 69.6 | 65.0 | 76.2 | 0.0006 |
|  |  |  |  | V | 18 | 23 | 1.000 | 0.914-1.000 | <0.0001 | ≤21.0 | 100.0 | 100.0 | 100 | 100.0 | - |
|  |  |  | 2 | APTT | 21 | 23 | 0.623 | 0.464-0.765 | 0.1607 | >35.6 | 33.3 | 95.7 | 87.5 | 61.1 | 0.0216 |
|  |  |  |  | V | 21 | 23 | 0.864 | 0.727-0.949 | <0.0001 | ≤26.3 | 81.0 | 91.3 | 89.5 | 84.1 | - |
|  | UFH | 150 IU/kg 3x a day | 1 | APTT | 58 | 93 | 0.755 | 0.678-0.821 | <0.0001 | >33.6 | 72.4 | 72.0 | 61.7 | 80.7 | 0.0546 |
|  |  |  |  | V | 58 | 93 | 0.849 | 0.782-0.902 | <0.0001 | ≤18.7 | 72.4 | 100.0 | 100 | 85.3 | - |
|  |  |  | 2 | APTT | 88 | 93 | 0.685 | 0.611-0.751 | <0.0001 | >30.7 | 72.7 | 59.1 | 62.7 | 69.6 | <0.0001 |
|  |  |  |  | V | 88 | 93 | 0.850 | 0.789-0.898 | <0.0001 | ≤18.5 | 69.3 | 100.0 | 100.0 | 77.5 | - |
| 2 | LMWH | 3000-4000 IU 1x a day | 1 | APTT | 35 | 41 | 0.611 | 0.493-0.721 | 0.0893 | >33.0 | 80.0 | 48.8 | 57.2 | 74.0 | <0.0001 |
|  |  |  |  | V | 35 | 41 | 0.897 | 0.930-1.000 | <0.0001 | <21.0 | 91.4 | 100.0 | 100.0 | 93.2 | - |
|  |  |  | 2 | APTT | 35 | 41 | 0.545 | 0.440-0.647 | 0.4494 | >36 | 49.1 | 65.9 | 65.9 | 49.1 | 0.4159 |
|  |  |  |  | V | 35 | 41 | 0.616 | 0.511-0.713 | 0.0447 | ≤30.4 | 52.7 | 73.2 | 72.5 | 53.6 | - |
| 3 | UFH | 12000 IU/d | 1 | APTT | 41 | 44 | 0.644 | 0.533-0.754 | 0.0182 | >35.6 | 51.2 | 79.6 | 70.0 | 63.7 | 0.0010 |
|  |  |  |  | V | 41 | 44 | 0.851 | 0.757-0.919 | <0.0001 | ≤25.0 | 85.4 | 77.3 | 77.8 | 85.0 | - |

APTT – activated partial thromboplastin time; TD – thrombodynamics; UFH – unfractionated heparin; LMWH – low molecular weight heparin
