# Supplementary material for: Thrombodynamics—A new global hemostasis assay for heparin monitoring in patients under the anticoagulant treatment
Source: PLoS One. 2018 Jun 28;13(6):e0199900. doi: 10.1371/journal.pone.0199900 (PMC6023127; doi:10.1371/journal.pone.0199900)
Supplement: S4 Table — (DOCX) [file pone.0199900.s004.docx]

**S4 Table. Heparin sensitivity: anti-Xa activity and V in TD**

| **Group** | **Heparin type** | **Heparin dosage** | **Point #** | **Test** | **Positive group, n** | **Negative group, n** | **AUC** | **95% CI** | **P (AUC>0.5)** | **Cut-Off** | **Sensitivity** | **Specificity** | **Positive predictive value** | **Negative predictive value** | **P** |
| --- | --- | --- | --- | --- | --- | --- | --- | --- | --- | --- | --- | --- | --- | --- | --- |
| 1 | LMWH | 6000 IU 2x a day | 1 | Anti-Xa | 21 | 23 | 1.000 | 0.920-1.000 | <0.0001 | >0.11 | 100.0 | 100.0 | 100.0 | 100.0 | 1.0000 |
|  |  |  |  | V | 21 | 23 | 1.000 | 0.920-1.000 | <0.0001 | <21.0 | 100.0 | 100.0 | 100.0 | 100.0 | - |
|  |  |  | 2 | Anti-Xa | 22 | 23 | 0.896 | 0.769-0.967 | <0.0001 | >0.07 | 81.8 | 82.6 | 81.8 | 82.6 | 0.6071 |
|  |  |  |  | V | 22 | 23 | 0.871 | 0.737-0.952 | <0.0001 | ≤26.3 | 81.8 | 91.3 | 90.0 | 84.0 | - |
| 2 | LMWH | 3000-4000 IU 1x a day | 1 | Anti-Xa | 36 | 29 | 0.984 | 0.917-1.000 | <0.0001 | >0.12 | 94.4 | 100.0 | 100.0 | 93.5 | 0.9121 |
|  |  |  |  | V | 36 | 29 | 0.986 | 0.919-1.000 | <0.0001 | ≤21.0 | 91.7 | 100.0 | 100.0 | 90.6 | - |
|  |  |  | 2 | Anti-Xa | 34 | 29 | 0.522 | 0.392-0.649 | 0.7681 | >0 | 88.24 | 0.0 | 50.8 | 0 | 0.3061 |
|  |  |  |  | V | 34 | 29 | 0.629 | 0.498-0.748 | 0.0662 | ≤25 | 26.47 | 93.1 | 81.8 | 51.9 | - |

Anti-Xa – Anti-Xa activity; TD – thrombodynamics; LMWH – low molecular weight heparin
