# Supplementary material for: Thrombodynamics—A new global hemostasis assay for heparin monitoring in patients under the anticoagulant treatment
Source: PLoS One. 2018 Jun 28;13(6):e0199900. doi: 10.1371/journal.pone.0199900 (PMC6023127; doi:10.1371/journal.pone.0199900)
Supplement: S5 Table — (DOCX) [file pone.0199900.s005.docx]

**S5 Table. Heparin sensitivity: ETP in TGT and V in TD**

| **Group** | **Heparin type** | **Heparin dosage** | **Point #** | **Test** | **Positive group, n** | **Negative group, n** | **AUC** | **95% CI** | **P (AUC>0.5)** | **Cut-Off** | **Sensitivity** | **Specificity** | **Positive predictive value** | **Negative predictive value** | **P** |
| --- | --- | --- | --- | --- | --- | --- | --- | --- | --- | --- | --- | --- | --- | --- | --- |
| 1 | UFH | 150 UI/kg 3x a day | 1 | ETP | 50 | 54 | 0.826 | 0.740-0.894 | <0.0001 | ≤782 | 64.0 | 88.9 | 84.2 | 72.7 | 0.6187 |
|  |  |  |  | Amax | 50 | 54 | 0.852 | 0.776-0.928 | <0.0001 | ≤111 | 78 | 85.19 | 83 | 80.69 | 0.2695 |
|  |  |  |  | Tmax | 50 | 54 | 0.771 | 0.680-0.862 | <0.0001 | >9 | 58 | 58 | 85.3 | 69.98 | 0.5448 |
|  |  |  |  | LagT | 50 | 54 | 0.718 | 0.616-0.819 | <0.0001 | >2.3 | 61.22 | 77.78 | 71.86 | 68.4 | 0.236 |
|  |  |  |  | V | 50 | 54 | 0.802 | 0.713-0.874 | <0.0001 | ≤19.9 | 70.0 | 98.2 | 97.2 | 77.9 | - |
|  |  |  | 2 | ETP | 50 | 54 | 0.641 | 0.541-0.733 | 0.0097 | ≤916 | 50.0 | 74.1 | 64.1 | 64.5 | 0.0086 |
|  |  |  |  | Amax | 50 | 54 | 0.67 | 0.566-0.773 | 0.0014 | ≤138.67 | 62 | 66.67 | 63.29 | 65.43 | 0.0212 |
|  |  |  |  | Tmax | 50 | 54 | 0.58 | 0.471-0.690 | 0.1505 | >6.8 | 64 | 51.85 | 55.19 | 60.85 | 0.0004 |
|  |  |  |  | LagT | 50 | 54 | 0.542 | 0.431-0.653 | 0.4554 | ≤2.76 | 92 | 18.52 | 51.13 | 71.41 | 0.0008 |
|  |  |  |  | V | 50 | 54 | 0.800 | 0.810-0.872 | <0.0001 | ≤19.6 | 66.0 | 98.2 | 97.1 | 75.7 | - |
| 2 | LMWH | 3000-4000 IU 2x a day | 1 | ETP | 10 | 17 | 0.853 | 0.664-0.959 | <0.0001 | ≤1155 | 100.0 | 58.8 | 58.8 | 100 | 0.0433 |
|  |  |  |  | Amax | 10 | 17 | 0.941 | 0.860-1.000 | <0.0001 | ≤151 | 90 | 88.24 | 81.8 | 93.75 | 0.1568 |
|  |  |  |  | Tmax | 10 | 17 | 0.876 | 0.691-1.000 | 0.0001 | >5.9 | 90 | 82.35 | 74.97 | 93.34 | 0.1919 |
|  |  |  |  | LagT | 10 | 17 | 0.835 | 0.667-1.000 | 0.0001 | >2.67 | 80 | 88.24 | 79.98 | 88.25 | 0.0546 |
|  |  |  |  | V | 10 | 17 | 1.000 | 0.872-1.000 | <0.0001 | ≤21.0 | 100.0 | 100.0 | 100.0 | 100.0 | - |
|  |  |  | 2 | ETP | 33 | 17 | 0.504 | 0.359-0.649 | 0.9610 | ≤1011 | 18.2 | 64.7 | 50.0 | 28.9 | 0.3626 |
|  |  |  |  | Amax | 33 | 17 | 0.569 | 0.398-0.739 | 0.4302 | >313 | 27.27 | 100 | 100 | 41.46 | 0.7166 |
|  |  |  |  | Tmax | 33 | 17 | 0.623 | 0.447-0.799 | 0.1712 | >5.4 | 60.61 | 76.47 | 83.33 | 50 | 0.9508 |
|  |  |  |  | LagT | 33 | 17 | 0.676 | 0.522-0.829 | 0.0253 | >2.83 | 27.27 | 100 | 100 | 41.4 | 0.6286 |
|  |  |  |  | V | 33 | 17 | 0.616 | 0.468-0.750 | 0.1587 | ≤30.4 | 48.5 | 82.4 | 84.2 | 45.2 | - |
| 3 | UFH | 12000 IU/d | 1 | ETP | 23 | 27 | 0.813 | 0.678-0.909 | <0.0001 | ≤1275 | 73.9 | 77.8 | 73.9 | 77.8 | 0.3746 |
|  |  |  |  | Amax | 23 | 27 | 0.804 | 0.677-0.930 | <0.0001 | ≤169.45 | 65.22 | 85.19 | 45.62 | 92.78 | 0.1505 |
|  |  |  |  | Tmax | 23 | 27 | 0.618 | 0.457-0.780 | 0.1512 | >6.8 | 60.87 | 62.96 | 58.33 | 65.38 | 0.0009 |
|  |  |  |  | LagT | 23 | 27 | 0.502 | 0.347-0.658 | 0.9757 | >0.05 | 95.65 | 11.11 | 47.83 | 74.99 | < 0.0001 |
|  |  |  |  | V | 23 | 27 | 0.859 | 0.732-0.941 | <0.0001 | ≤25.0 | 78.3 | 92.6 | 90.0 | 83.3 | - |
| ETP – endogenous thrombin potential; TGT – thrombin generation test; TD – thrombodynamics; UFH – unfractionated heparin; LMWH – low molecular weight heparin | | | | | | | | | | | | | | | |
