# Supplementary material for: Thrombodynamics—A new global hemostasis assay for heparin monitoring in patients under the anticoagulant treatment
Source: PLoS One. 2018 Jun 28;13(6):e0199900. doi: 10.1371/journal.pone.0199900 (PMC6023127; doi:10.1371/journal.pone.0199900)
Supplement: S6 Table — (DOCX) [file pone.0199900.s006.docx]

**S6 Table. Heparin sensitivity: alpha in TEG and V in TD**

| **Group** | **Heparin type** | **Heparin dosage** | **Point #** | **Test** | **Positive group, n** | **Negative group, n** | **AUC** | **95% CI** | **P (AUC>0.5)** | **Cut-Off** | **Sensitivity** | **Specificity** | **Positive predictive value** | **Negative predictive value** | **P** |
| --- | --- | --- | --- | --- | --- | --- | --- | --- | --- | --- | --- | --- | --- | --- | --- |
| 2 | LMWH | 3000-4000 IU 1x a day | 1 | R | 23 | 33 | 0.592 | 0.437-0.747 | 0.2443 | >13.6 | 82.61 | 54.55 | 55.91 | 81.8 | < 0.0001 |
|  |  |  |  | k | 23 | 33 | 0.642 | 0.490-0.793 | 0.0673 | >3.8 | 95.65 | 45.45 | 55.03 | 93.74 | < 0.0001 |
|  |  |  |  | alpha | 23 | 33 | 0.861 | 0.742-0.939 | <0.0001 | ≤29.3 | 65.2 | 93.9 | 88.2 | 75.9 | 0.0057 |
|  |  |  |  | MA | 23 | 33 | 0.526 | 0.371-0.681 | 0.7391 | ≤58.6 | 78.26 | 48.48 | 51.45 | 76.17 | < 0.0001 |
|  |  |  |  | V | 23 | 33 | 0.999 | 0.934-1.000 | <0.0001 | ≤24.0 | 100.0 | 97.0 | 95.8 | 100.0 | - |
|  |  |  | 2 | R | 41 | 33 | 0.638 | 0.511-0.765 | 0.0327 | >41 | 46.34 | 78.79 | 73.07 | 54.17 | 0.7932 |
|  |  |  |  | k | 41 | 33 | 0.575 | 0.435-0.714 | 0.2954 | >2.6 | 92.68 | 33.33 | 63.32 | 78.57 | 0.6977 |
|  |  |  |  | alpha | 41 | 33 | 0.561 | 0.441-0.676 | 0.3861 | ≤35.5 | 63.4 | 57.6 | 65.0 | 55.9 | 0.5774 |
|  |  |  |  | MA | 41 | 33 | 0.538 | 0.403-0.674 | 0.5774 | >33 | 100 | 15.15 | 59.41 | 100 | 0.4274 |
|  |  |  |  | V | 41 | 33 | 0.613 | 0.493-0.724 | 0.0884 | ≤30.4 | 48.8 | 84.9 | 80.0 | 57.1 | - |
| 3 | UFH | 12000 IU/d | 1 | R | 38 | 42 | 0.724 | 0.609-0.839 | 0.0001 | >16.9 | 73.68 | 73.81 | 71.79 | 75.61 | 0.0146 |
|  |  |  |  | k | 38 | 42 | 0.793 | 0.688-0.898 | <0.0001 | >6.2 | 73.68 | 85.71 | 82.35 | 78.26 | 0.1319 |
|  |  |  |  | alpha | 38 | 42 | 0.777 | 0.667-0.886 | <0.0001 | ≤31.6 | 73.68 | 85.71 | 82.35 | 78.26 | 0.0641 |
|  |  |  |  | MA | 38 | 42 | 0.68 | 0.562-0.797 | 0.0027 | ≤56.1 | 52.63 | 76.19 | 66.67 | 64 | 0.0021 |
|  |  |  |  | V | 39 | 42 | 0.867 | 0.774-0.932 | <0.0001 | ≤25.0 | 84.6 | 81.0 | 80.5 | 85.0 | - |

TEG - thromboelastography; TD – thrombodynamics; UFH – unfractionated heparin; LMWH – low molecular weight heparin
