# Supplementary material for: Thrombodynamics—A new global hemostasis assay for heparin monitoring in patients under the anticoagulant treatment
Source: PLoS One. 2018 Jun 28;13(6):e0199900. doi: 10.1371/journal.pone.0199900 (PMC6023127; doi:10.1371/journal.pone.0199900)
Supplement: S7 Table — (DOCX) [file pone.0199900.s007.docx]

**S7 Table. Comparison of the hemostasis assays’ sensitivity to heparins**

| **Parameter** | **Therapy** | | | | **Prophylaxis** | | |
| --- | --- | --- | --- | --- | --- | --- | --- |
|  | **UFH** | | **LMWH** | | **UFH** | **LMWH** | |
|  | **Point 1** | **Point 2** | **Point 1** | **Point 2** | **Point 1** | **Point 1** | **Point 2** |
| V in TD | High | High | High | High | High | High | Low |
| APTT | Moderate | Low | Low | Low | Low | Low | Low |
| Anti-Xa assay | - | - | High | High | - | High | Low |
| Amax in TGT | High | Low | - | - | High | High | Low |
| Alpha in TEG | - | - | - | - | Moderate | High | Low |

AUC < 0.7 – low sensitivity (Low); moderate when AUC ∈ [0.7- 0.8] – moderate sensitivity (Moderate); AUC > 0.8 – high sensitivity (High); “-“ – was not measured
