# Supplementary material for: Thrombodynamics—A new global hemostasis assay for heparin monitoring in patients under the anticoagulant treatment
Source: PLoS One. 2018 Jun 28;13(6):e0199900. doi: 10.1371/journal.pone.0199900 (PMC6023127; doi:10.1371/journal.pone.0199900)
Supplement: S8 Table — (DOCX) [file pone.0199900.s008.docx]

**S8 Table. ROC analysis with Bootstrap estimation (3000 random curves): APTT vs V in TD**

| **Group** | **Heparin type** | **Heparin dosage** | **Point #** | **Test** | **AUC** | **95% CI** | **Bootstrap AUC** | **Bootstrap 95% CI** |
| --- | --- | --- | --- | --- | --- | --- | --- | --- |
| 1 | LMWH | 6000 IU  2x a day | 1 | APTT | 0.720 | 0.558-0.849 | 0.713 | 0.583-0.835 |
|  |  |  |  | V | 1.000 | 0.914-1.000 | 1.000 | 1.000-1.000 |
|  |  |  | 2 | APTT | 0.623 | 0.464-0.765 | 0.625 | 0.481-0.760 |
|  |  |  |  | V | 0.864 | 0.727-0.949 | 0.835 | 0.720-0.983 |
|  | UFH | 150 UI/kg  3x a day | 1 | APTT | 0.755 | 0.678-0.821 | 0.748 | 0.681-0.812 |
|  |  |  |  | V | 0.849 | 0.782-0.902 | 0.835 | 0.768-0.900 |
|  |  |  | 2 | APTT | 0.685 | 0.611-0.751 | 0.676 | 0.610-0.740 |
|  |  |  |  | V | 0.850 | 0.789-0.898 | 0.842 | 0.786-0.891 |
| 2 | LMWH | 3000-4000 IU 1x a day | 1 | APTT | 0.611 | 0.493-0.721 | 0.606 | 0.500-0.703 |
|  |  |  |  | V | 0.987 | 0.930-1.000 | 0.983 | 0.930-1.000 |
|  |  |  | 2 | APTT | 0.545 | 0.440-0.647 | 0.537 | 0.441-0.634 |
|  |  |  |  | V | 0.616 | 0.511-0.713 | 0.613 | 0.424-0.805 |
| 3 | UFH | 12000 IU/d | 1 | APTT | 0.644 | 0.533-0.754 | 0.641 | 0.542-0.738 |
|  |  |  |  | V | 0.851 | 0.757-0.919 | 0.837 | 0.759-0.908 |

APTT – activated partial thromboplastin time; TD – thrombodynamics; UFH – unfractionated heparin; LMWH – low molecular weight heparin
