# Supplementary material for: Thrombodynamics—A new global hemostasis assay for heparin monitoring in patients under the anticoagulant treatment
Source: PLoS One. 2018 Jun 28;13(6):e0199900. doi: 10.1371/journal.pone.0199900 (PMC6023127; doi:10.1371/journal.pone.0199900)
Supplement: S9 Table — (DOCX) [file pone.0199900.s009.docx]

**S9 Table. Heparin treatment effects on hemostasis assays**

| **Group** | **Heparin type** | **Heparin dosage** | **Point** | **V** | | | **ETP** | | | **alpha** | | |
| --- | --- | --- | --- | --- | --- | --- | --- | --- | --- | --- | --- | --- |
|  |  |  |  | **hypo** | **normal** | **hyper** | **hypo** | **normal** | **hyper** | **hypo** | **normal** | **hyper** |
| 1 | LMWH | 6000 IU  2x a day | 1 | 5% | 95% | 0% | - | - | - | - | - | - |
|  |  |  | 2 | 36% | 50% | 14% | - | - | - | - | - | - |
|  | UFH | 150 UI/kg 3x a day | 1 | 77% | 18% | 5% | 74% | 24% | 2% | - | - | - |
|  |  |  | 2 | 70% | 22% | 8% | 46% | 49% | 5% | - | - | - |
| 2 | LMWH | 3000-4000 IU 1x a day | 1 | 78% | 19% | 3% | 50% | 50% | 0% | 43% | 48% | 9% |
|  |  |  | 2 | 7% | 37% | 56% | 5% | 70% | 25% | 9% | 81% | 10% |
| 3 | UFH | 12000 IU/d | 1 | 66% | 24% | 10% | 17% | 70% | 13% | 38% | 41% | 21% |

ETP – endogenous thrombin potential; UFH – unfractionated heparin; LMWH – low molecular weight heparin
